# Salinization of Alpine rivers during winter months

**DOI:** 10.1007/s11356-020-11077-4

**Published:** 2020-10-07

**Authors:** Georg H. Niedrist, Miguel Cañedo-Argüelles, Sophie Cauvy-Fraunié

**Affiliations:** 1 Department of Ecology, River and Conservation Research, University of Innsbruck, Innsbruck, Austria; 2 Freshwater Ecology, Hydrology and Management group (FEHM), Departament de Biologia Evolutiva, Ecologia i Ciències Ambientals, Institut de Recerca de l’Aigua (IdRA), Universitat de Barcelona, Barcelona, Spain; 3 INRAE, UR RIVERLY, Centre de Lyon-Villeurbanne, Villeurbanne Cedex, France

**Keywords:** Road salt, Alpine streams, Deicing, Land use, Water pollution, Temporal variation

## Abstract

Human-induced (i.e., secondary) salinization affects aquatic biodiversity and ecosystem functioning worldwide. While agriculture or resource extraction are the main drivers of secondary salinization in arid and semi-arid regions of the world, the application of deicing road salt in winter can be an important source of salts entering freshwaters in cold regions. Alpine rivers are probably affected by salinization, especially in highly populated mountain regions, although this remains to be explored. In this study, we analyzed multi-year conductance time series from four rivers in the European Alps and demonstrated that the application of deicing road salt is linked to peaking rivers’ salinity levels during late winter/early spring. Especially in small catchments with more urban surfaces close to the rivers, conductance increased during constant low-flow periods in late winter and was less correlated with discharge than in summer. Thus, our results suggest that small rivers highly connected to urban infrastructures are prone to considerable salinity peaks during late winter/early spring. Given the low natural level of salinities in Alpine rivers, the aquatic biodiversity might be significantly affected by the recorded changes in conductance, with potential consequences on ecosystem functioning. Thereby, we urge the research community to assess the impact of secondary salinization in Alpine rivers and call for an implementation of management practices to prevent the degradation of these pristine and valuable ecosystems.

**Electronic supplementary material:**

The online version of this article (10.1007/s11356-020-11077-4) contains supplementary material, which is available to authorized users.

## Introduction

Freshwater habitats (e.g., lakes, rivers, streams, wetlands) are threatened by secondary (i.e., anthropogenic) salinization worldwide (Kaushal et al. 2018; Cañedo-Argüelles et al. 2018). For a long time, freshwater salinization has been mostly related to agriculture and pasture in arid and semi-arid lowland regions (Cañedo-Argüelles 2020). However, different studies have shown that lakes (Dugan et al. 2017) and streams (Peters and Turk 1981; Godwin et al. 2003; Kaushal et al. 2005) are becoming increasingly saltier due to the application of road salt to improve driving safety during winter months. Yet, the salinization of mountain freshwater ecosystems has been almost exclusively investigated in the USA, and it is still poorly understood. A wider characterization and quantification of freshwater salinization in mountain areas is urgently needed because it could be affecting species survival (Crowther and Hynes 1977; Collins and Russell 2009; Corsi et al. 2010) and fitness (Karraker 2007; Hintz and Relyea 2019), as well as ecosystem functioning and services (Millenium Ecosystem Assessment 2005; Herbert et al. 2015; Hintz and Relyea 2019).

In Alpine areas, secondary salinization has not yet been perceived as a major problem due to the generally low concentration of dissolved ions in most meltwater streams (e.g., Brown et al. 2006; Niedrist and Füreder 2016). However, in these areas, anthropogenic inputs can disproportionally increase baseline salinities (Olson 2019), and the relative chloride concentration compared with pre-polluted situations (e.g., chloride concentration in a tributary of Mirrow Lake increased > 100 times from ~ 0.7 to ~ 80 mg Cl L^−1^ within 25 years (Likens and Buso 2009), equaling an estimated increase in conductance from 30 to 3400 μs cm^−1^). The ecological effect of relative increases in salinity needs to be considered within the context of biodiversity conservation in Alpine freshwaters, since aquatic organisms in these ecosystems have evolved under relatively low salinities (Niedrist and Füreder 2016) and could be particularly sensitive to salinization (Kefford et al. 2016). Indeed, laboratory studies showed that aquatic invertebrates coming from low-conductivity rivers were more sensitive to salt pollution than the same invertebrates from higher-conductance rivers (Clements and Kotalik 2016). This result suggests that the relative deviations from the natural background level need to be assessed in Alpine and mountain ecosystems.

The European Alps are a mountain region that relies on tourism (e.g., skiing resorts) and agriculture for economic development. During winter, snowfall and cold conditions can cause icy roads. In most regions of the European Alps, the use of deicing salts to maintain clear road and other urban pavements free of ice during winter is a common practice. For example, in the Alpine region of Tyrol, Austria, an average of 30,000 tons of salt (sodium chloride) are applied annually to approximately 2240 km of roads (Amt der Tiroler Landesregierung, press release). During periods of snow-removal from the roads (which is partly poured into rivers and stored next to them), and especially during melting and precipitation events, these salts might enter the rivers, as it has been shown for a northern Italian catchment (Nava et al. 2020) and North American (Crowther and Hynes 1977; Dugan et al. 2020) and Scandinavian streams (Ruth 2003). In all the cases, seasonal salinity cycles were synchronized with road salt applications. Nevertheless, the magnitude, seasonal patterns, and short-term variabilities of secondary salinization in Alpine rivers remain unknown. Obtaining this information is crucial to preserve Alpine river networks, which are largely unimpacted aquatic ecosystems and important biodiversity hotspots (Khamis et al. 2014). Moreover, the impact of salinization in these mountain areas could become stronger in the future due to steady increases in water demand associated with tourism and urbanization (Beniston 2012; Klug et al. 2012). Accordingly, the primary objectives of this study were to (i) define temporal trends of salinity in a selected Alpine stream based on long-term records of specific conductance, and to (ii) compare conductance patterns among seasons and between years in four different catchments in the European Alps with varying degrees of urbanization.

## Material and methods

### Study sites

**Fig. 1:**
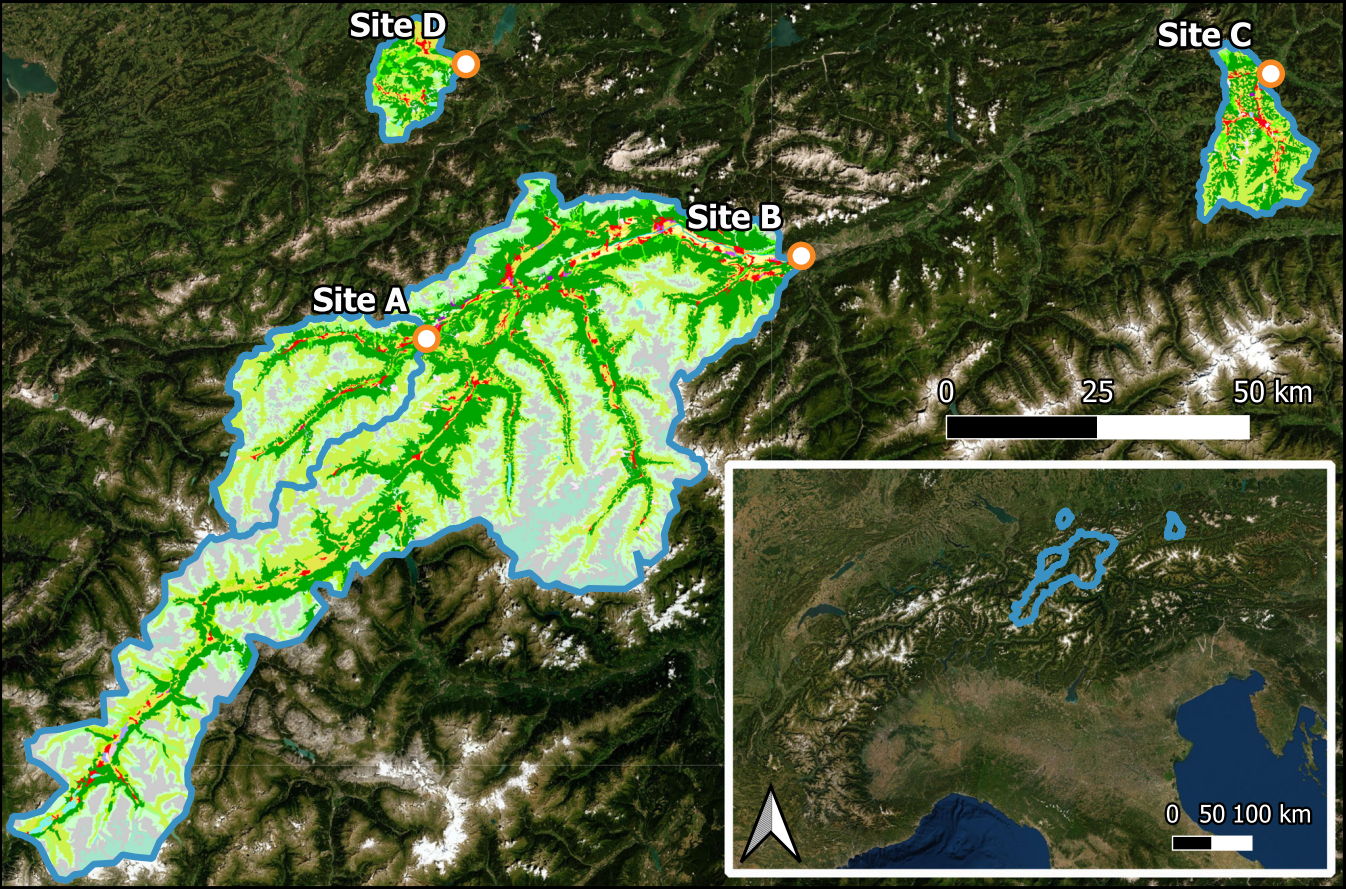
Study sites and catchments in the European Alps with indicated CORINE land cover types (red = urban areas, greens = forest, white/gray = sparsely vegetated areas/rocks, light blue = glaciers and perpetual snow)

The studied sites are large Alpine rivers in the region of Tyrol, Austria (Eastern Central Alps), that drain catchments with and without glacial influence (Fig. 1). The monitoring stations are located at an elevation of 777 m (Sanna, site A), 571 m (Inn, site B), 660 m (Kitzbueheler Ache, site C), and 807 m a.s.l. (Vils, site D), integrating water properties of streams draining catchment areas of 708 km^2^ (site A), 5243 km^2^ (site B), 327 km^2^ (site C), and 198 km^2^ (site D), respectively. The catchment geology of sites A, B, and C is dominated by non-limestone bedrock, while stream D drains a catchment with calcareous bedrock (Brandner 1980). Besides the size of catchment areas, the monitored streams differ in their water source contributions. While all streams are spring and snow fed, streams A and B have additional contributions from glaciers. Mean annual discharge was 20.2 m^3^/s (1983–2015) at site A, 11.3 m^3^/s at site C (1951–2015), and 7.6 m^3^/s at site D, while it was much higher (165 m^3^/s) at site B (1971–2015) (BMNT Abteilung Wasserhaushalt 2018).

### Data sources

Mean daily discharge data were obtained from the Hydrographisches Jahrbuch 2015 (BMNT Abteilung Wasserhaushalt 2018). Catchments were delineated using QGIS (QGIS Development Team 2009) with the GRASS GIS add-on (GRASS Development Team 2017), and ratios of anthropogenic and glacial land cover were obtained from freely available CORINE Land Cover (CLC) data. Digital elevation data (retrieved from the EU-funded COPERNICUS platform) had a 25-m resolution.

We used records of electrical conductance to indicate salinity patterns in the studied streams. Electrical conductivity (in Siemens per centimeter) can often be used as a proxy for the concentration of dissolved ions (e.g., Williams and Sherwood 1994; Corsi et al. 2010; Griffith 2014), since the ionic activity largely determines the ability of a solution to transmit electrical current. However, as conductivity also depends on water temperature, the temperature-corrected electrical conductivity (temperature-corrected for 25 °C), expressed as specific conductance, is preferably used as indicator of water quality, and to investigate biological processes (Hayashi et al. 2012).

Continuous data were obtained from the automatic gauging stations along the rivers Sanna, Inn, and Kitzbueheler Ache, which monitor specific conductance of the water at 15-min intervals and discharge as daily average. The monitoring station at site A has been running since May 2012, and records at site B, site C, and site D started in March 2017, in April 2017, and in May 2018, respectively.

### Analysis and statistics

Relative land use in each of the catchments was aggregated for the following categories: urban surfaces, agricultural areas, semi-natural areas (including forest and grassland), wetlands, water bodies, and glaciers.

The high-frequency data of conductance and discharge were used to calculate monthly mean time series (*ts* function). Consequently, a “Seasonal and Trend decomposition using Loess” (STL) was performed (Cleveland et al. 1990) to decompose the monthly time series into long-term trends (using a 12-month moving average), seasonal cycles, and random (non-cyclic) variations. Simple linear models coupled with F-tests were then used to describe and verify the overall trend of water conductance and discharge after having removed seasonal cycles and random residuals. For this, we considered relationships as significant, when the probability of alpha-error was below 5% (*α* = 0.05). We performed regression diagnostics to check model performance (normal distribution and homoscedasticity of residuals, no influential observation). Such time series decomposition was only possible for site A (2012–2019), while records from the other sites were too short. Within years, we discriminated between winter and summer periods. “Winter” was defined as the period when winter tires were mandatory for cars in the region (November 1 until April 15), while “summer” was set as the period with high meltwater runoff (May 15 until September 15). The discrepancy in specific conductance between these seasons was expressed in absolute and relative differences. Cross-correlation on previously pre-whitening time series was used to assess the relationship between discharge and conductivity (Dean and Dunsmuir 2016) in addition to conductance–discharge plots (similar to concentration-discharge plots (Godsey et al. 2009; Moatar et al. 2017). All plots and analyses were performed in the R v3.6.1 (R Core Team 2018) using the packages *timeSeries* (Wuertz et al. 2017), *tseries* (Trapletti and Hornik 2019), *TSA* (Cryer and Chan 2008), and *ggpubr* (Kassambara 2018).

Published chloride concentrations were converted to equivalent conductivities of theoretical solutions as follows:

$$ \left(\mathrm{equivalent}\right)\ \mathrm{conductivity}\ \left(\frac{\upmu \mathrm{s}}{\mathrm{cm}}\right)=\frac{\mathrm{salt}\ \mathrm{concentration}\left(\frac{\mathrm{g}}{\mathrm{L}}\right)\ast \mathrm{molar}\ \mathrm{ionic}\ \mathrm{conductivity}\ \left(\frac{\mathrm{S}}{\mathrm{m}}\ \mathrm{per}\frac{\mathrm{m}\mathrm{ol}}{\mathrm{L}}\right)}{\mathrm{m}\mathrm{ol}\mathrm{ar}\ \mathrm{mass}\ \left(\frac{\mathrm{g}}{\mathrm{m}\mathrm{ol}}\right)}\ast 10000 $$

## Results

### Main land use in catchments

Semi-natural areas (forest and grassland) was identified as the dominant land cover type in all catchments (73–92%). The catchments of sites C and D had higher proportions of non-natural areas, with 18% and 20.4% of agricultural surfaces, and 7.9% and 5.7% of urban surfaces for sites C and D, respectively (Table 1). In contrast to site C and D (both 0%), site A and site B had a low to moderate relative and absolute glacier-coverage in the catchments (0.9%/6 km^2^ and 3.5%/185 km^2^, respectively). Given its small size, and contrary to relative differences, site D had the smallest catchment area covered by urban surfaces (Table 1). River C (medium to large catchment size) and in particular river A (medium catchment size) flow close to urban areas (including skiing area facilities and high traffic close to the water), while river B drains a large catchment area and at site D the urban land cover in the small catchment is not close to the water.

**Table 1:** Major land cover types, their relative and absolute occurrence in the four investigated hydrological catchments, and mean (m) and standard error (sd, 68% CI) of daily mean specific conductance levels of study rivers at site A, site B, site C, and site D, including the coordinates (latitude, longitude, WGS 84). ‘99th percentiles’ indicates the highest values in these rivers (without outliers)

	Site A		Site B		Site C		Site D	
Coordinates	47.518, 12.417		47.262, 11.381		47.519, 12.418		47.551, 10.651	
Land cover category	%	km^2^	%	km^2^	%	km^2^	%	km^2^
Agriculture	4.0	28.4	6.1	322.4	18.8	61.4	20.4	40.1
Urban landscape	3.5	24.9	3.0	155.1	7.9	25.7	5.7	11.2
Semi-natural areas	91.5	646.5	87.0	4561	73.2	239.5	73.0	143.4
Glaciers and perpetual snow	0.9	6.2	3.5	184.7	0.0	0	0.0	0
Inland waters	0.0	0.3	0.3	16.6	0.1	0.3	0.7	1.3
Wetlands	0.0	0.3	0.1	4.1	0.1	0.3	0.1	0.3
Electrical conductance (μs cm^−1^)								
99th percentiles	232.5		359.6		332.0		459.2	
	Mean	sd	Mean	sd	Mean	sd	Mean	sd
Overall	178.3	25.8	222.9	86.2	247.4	50.1	414.9	39.9
Summer	159.2	23.5	142.9	43.8	237.8	44.5	421.1	22.8
Winter	192.5	21.0	292.3	44.4	256.2	53.2	410.2	47.2

### Fluctuations in specific conductance

Overall, the conductance based on multiannual trend at site A, the site with the longest records, increased significantly from 2012 to 2019 (F_1,61_ = 21.1, *R*^2^ = 0.26, *p* < 0.001) for 11 μs cm^−1^, while long-term discharge showed a non-significant decrease (F_1,70_ = 3, *R*^2^ = 0.04, *p* = 0.088, Fig. S1). The comparison of different resolutions of this time series illustrates the different variability of the data. The high-frequency fluctuations that occur during winter have not been recorded in summer (Fig. S2).

**Fig. 2:**
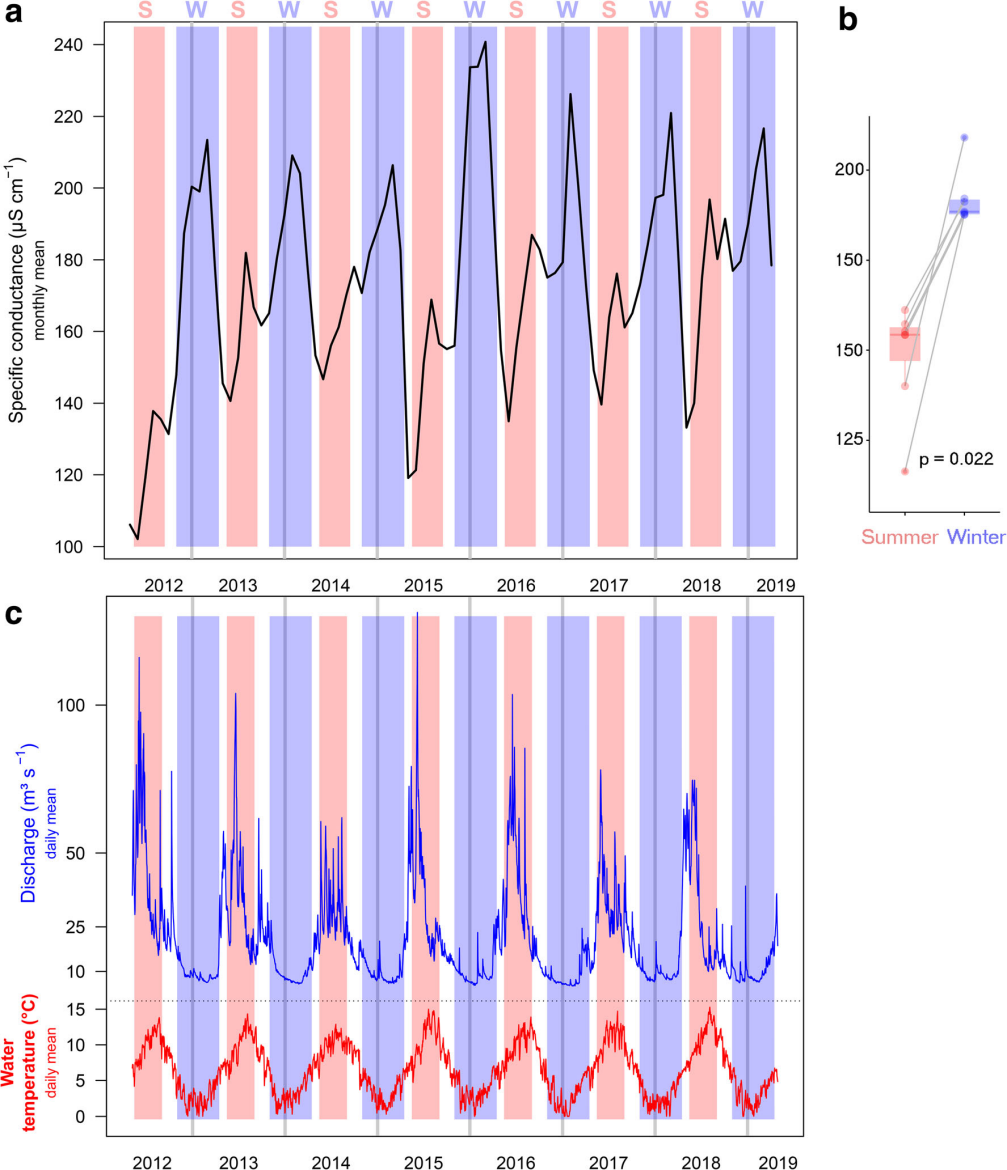
Monthly mean-specific conductance (**a**), its pairwise comparison (including pairwise *t* test, where each pair is from 1 year) and overall comparison between season-specific means per year (summer vs. winter, **b**), and daily mean discharge and water temperature patterns (**c**) at site A, the river Sanna in Tyrol, Central Alps. Blue and red areas mark high-flow conditions during summer (May 15 until September 15, “S”) and periods where winter tires are mandatory for cars in the region (November 1 until April 15, “W”). Dark vertical lines indicate the beginning of each year

**Fig. 3:**
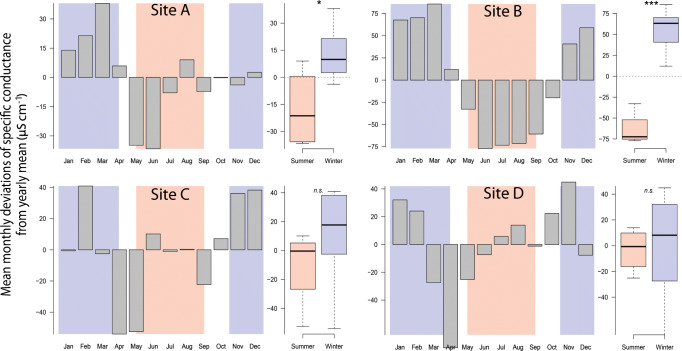
Month-specific deviations from multi-year trend (moving average) of specific conductance in μs cm^−1^ between April 2017 and April 2019 (from June 2018 to April 2019 for site D) in different streams (site A–site D) in each left panel, and the summarized difference of deviations between the seasons “Summer” (May to August in red), and “Winter” (November–April in blue) in each right panel

Overall, mean monthly conductance at site A ranged from 102.1 to 240.8 μs cm^−1^, with a mean level of 173.1 μs cm^−1^ and an interquartile range of measured data from 155.0 to 191.7 μs cm^−1^ from May 2012 to April 2019 (Fig. 2a). During the study period, mean monthly conductivity was significantly higher during winter compared with summer (Fig. 2b). In all monitored years, two main peaks in mean monthly conductance occurred: one during late winter (i.e., between February and March), with the highest winter value, 243.1 μs cm^−1^, observed at the end of March 2016, and a smaller one during summer, with the highest summer values varying between 139.5 μs cm^−1^ (August 2012) and 196.8 μs cm^−1^ (August 2018). After removing the multi-year trend and averaging monthly differences of all the studied years, we found that electrical conductance was up to 38.0 μs cm^−1^, 85.7 μs cm^−1^, or 40.9 μs cm^−1^ higher than the running average in March or February for sites A, B, and C, respectively. In contrast, the highest positive deviation of mean monthly conductance occurred in November at site D (44.9 μs cm^−1^ above running average) and the highest negative in April (with − 74.3 μs cm^−1^ below running average). Mean monthly deviations during summer were low or negative in all streams, with the highest negative deviations observed between April and June, depending on the study site (Fig. 3). In addition, we observed a seasonal pattern in both discharge and temperature time series, with highest discharges and temperatures recorded during summer (Fig. 2c). Conductance in sites B, C, and D also showed a seasonal pattern with the highest values during winter, and the lowest during summer.

### Specific conductance and rivers’ discharge

**Fig. 4:**
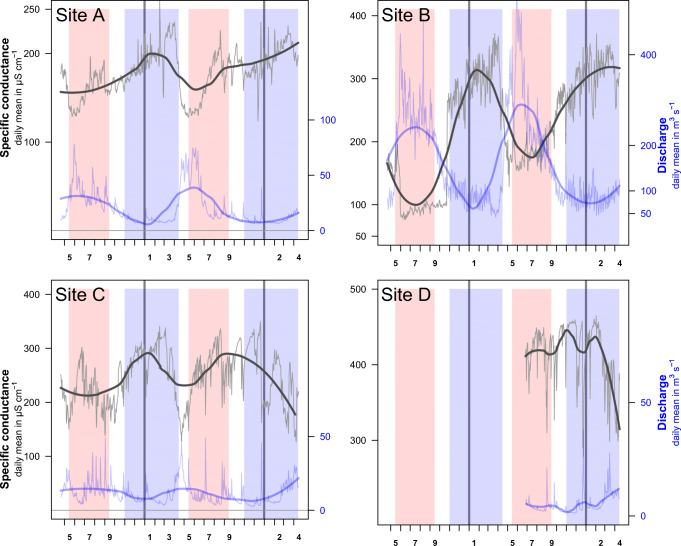
Yearly pattern of electrical conductance and discharge in all study sites (site A–site D) from spring 2017 to spring 2019. Blue and red areas mark high-flow conditions during summer (May 15 until September 15) and periods where winter tires are mandatory for cars in the region (November 1 until April 15). Lines denote daily measurements of conductance (in gray) and discharge (in blue), while bold red lines are moving averages fitted using loess method (span = 0.5). Numbers (*x*-axis) indicate calendar months. For overall conductance levels of each site, see Table 1

**Fig. 5:**
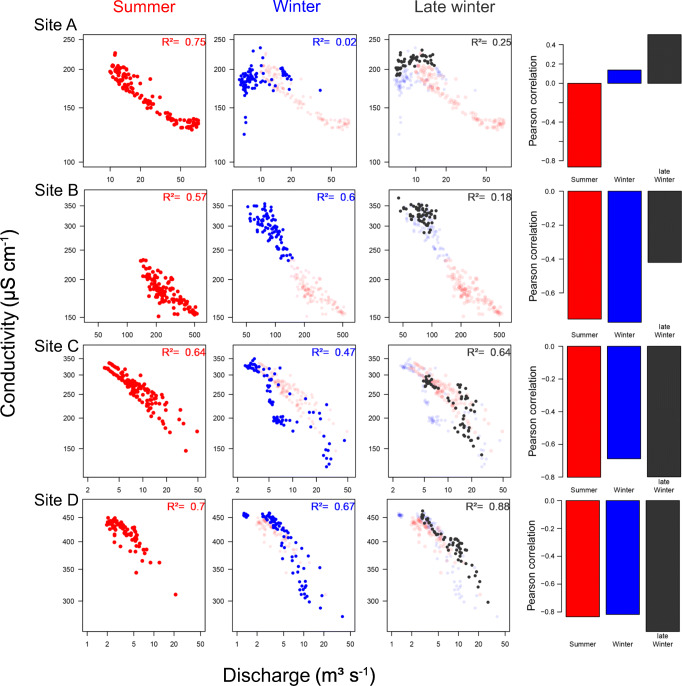
Season-specific (summer in red and winter in blue) discharge–conductance plots in all study sites (site A–site D) for one hydrological year (05.2018–04.2019) with indication of the quality of the relationship (*R*^2^) and the correlation (Pearson correlation). Since conductance is usually negatively related to discharge (see also Brown et al. (2006) for mountain streams), dissimilar correlations in summer and in winter (and late winter) indicate additional influences on ion balance, apart from discharge variabilities

Generally, conductance was negatively related with discharge. Between May 2017 and April 2019 for sites A, B, and C, and between June 2018 and April 2019 for site D, cross-correlation coefficients between discharge and conductivity at lag 0 were − 0.21, − 0.39, − 0.68, and − 0.75, respectively (*p* < 0.05). Conductance and discharge exhibited antagonist seasonal fluctuations, with high discharge and low conductance during summer in most streams, and vice versa during winter (Fig. 4). However, within the winter months characterized by constant low-flows and the absence of fluctuations in discharge, we observed considerable increases and peaks in conductance, especially towards the end of winter in February and March most clearly at sites A and C, but also at site B (Fig. S3). In contrast, no clear discrepancies in the relationship between discharge and conductivity were observed at site D for summer and winter (Fig. 5 and Fig. S3). When comparing the cross-correlation between conductivity and discharge across seasons (based on the time series subsets from summer 2018 to winter 2018/2019, Fig. S3) and the discharge-conductance relationships of one hydrological year (Fig. 5), both variables correlated more during summer than during winter for sites A, B, and C (− 0.42 vs. − 0.24, − 0.64 vs. − 0.58, and − 0.78 vs. − 0.51 for summer vs. winter), while it was similar for both periods for site D (− 0.70 vs. − 0.77, and Fig. S3).

## Discussion

We observed a clear seasonal pattern in electrical conductance in some of the studied streams, characterized by increases at the end of the winter period. Seasonal changes in electrical conductance in Alpine rivers could be attributed to the temporally varying contribution of different water sources and quantities to mountain streams, especially in catchments with glaciers (Gurnell and Fenn 1985; Dzikowski and Jobard 2012). However, the high conductance at the end of the winter period is most likely related to artificial salt inputs in those rivers, where the usually strong relationship between electrical conductance and discharge during summer has been found to be weaker and partly reversed during winter and late winter. This is particularly pronounced in catchments, where well-used urban structures are close to the rivers. However, general quantifications of urban land cover in the whole catchments could not explain these patterns, as found in similar assessments on river pollution studies (Shen et al. 2015). Despite the low or absence of fluctuations in discharge, we observed important changes in conductance during the winter period, while changes during summer were negatively related to changes in discharge. Thus, the observed high conductance levels at the end of the deicing salt application period (especially in catchments with dense urban surface areas close to the river), also corresponding to the melting season of accumulated snow and salt besides roads and streams, suggest that road salt governs the observed trends in Alpine watersheds with considerable connectivity to urban structures. In addition, the effect of salt input during winter on the aquatic habitats might also be reinforced by the lower runoff and the associated lower dilution in this Alpine dry season. Indeed, when comparing streams of different catchment areas and different discharges, we found that the annual variability in conductance, in particular the differences between summer and late winter, was greater in small streams, where dilution of pollutants is minimal (Williams and Melack 1997).

Besides this summer-winter pattern, our temporal data indicates that the baseline of specific conductance significantly increases over time, aligning with the global trends in freshwater ecosystems (e.g., Kaushal et al. 2005, 2018; Estévez et al. 2019; Le et al. 2019). Although agriculture (Williams 2001; Cañedo-Argüelles et al. 2013) and resource extraction (Palmer et al. 2010; Cañedo-Argüelles et al. 2012; Vidic et al. 2013) are probably the main drivers of salinization worldwide, this study provides further evidence that it is very likely that the main salt input for freshwater ecosystems in mountainous areas comes from the roads or other urban structures (Swinton et al. 2015; Corsi et al. 2015; Hintz and Relyea 2017; Nava et al. 2020). The observed levels and seasonal changes were relatively small compared with other studied aquatic systems outside the Alps (Kaushal et al. 2005; Corsi et al. 2010). However, it should be noticed that the relative urban landscapes in the study catchments were very low (ranging from 3.0 to 7.9%).

Increasing salinity levels might have consequences for the fitness of different aquatic organisms (Hintz and Relyea 2019; Cañedo-Argüelles 2020). Until a certain level, increasing salinity might have positive effects on the physiology of some species due to decreased osmoregulation activity (Kefford et al. 2016). However, aquatic biodiversity, including bacteria and fungi (Gonçalves et al. 2018), aquatic invertebrates (Cañedo-Argüelles et al. 2016), fish (Hintz and Relyea 2017), and amphibians (Karraker et al. 2008) can be significantly reduced when certain salinity level is exceeded. For example, elevated chloride concentrations (> 1000 mg/L, which equals an estimated conductivity of 2172 μs cm^−1^) in streams at much higher levels than reported here can reduce the biomass of autotrophic standing crops and the diversity of benthic algae and invertebrates (e.g., Demers and Sage 1990; Corsi et al. 2010). Besides directly reducing populations of sensitive taxa through mortality, lower salinization events can affect population fitness through sub-lethal effects. For example, salinization has been shown to reduce the growth of mayflies (Hassell et al. 2006) and fish, especially in their early life stages (Hintz and Relyea 2017). All these impacts on aquatic biodiversity are especially relevant for mountain streams, since organisms in these streams have evolved under low salt concentrations and should have a lower tolerance to increased salt concentrations (Kefford et al. 2016). Concordantly, Kotalik et al. (2017) showed how mountain stream invertebrates were significantly affected by salt concentrations lower than those recommended by the EPA. The short pulses of conductance during late winter observed in one of the streams (corresponding to ~ 51% of the multiannual monthly mean conductance) might ultimately stress aquatic biota (microbiota, algae, invertebrates) and thereby affect ecosystem processes in these systems (Blasius and Merritt 2002; Benbow and Merritt 2004; Cañedo-Argüelles et al. 2014; Hintz and Relyea 2019), with potential stronger effects than those caused by continuous salt pollution (Marshall and Bailey 2004).

Salinization can also increase the mobilization of other substances (e.g., heavy metals) in soils (Acosta et al. 2011; Schuler and Relyea 2018) or change biochemical processes, such as denitrification rates, nitrogen export, or release of DOC, nitrogen, and soluble reactive phosphorus (Herbert et al. 2015; Kaushal et al. 2019; Hintz and Relyea 2019), with indirect effect on aquatic ecosystems (Löfgren 2001; Bäckström et al. 2004). In times of hydrological and socio-economic changes in mountain regions (Huber et al. 2005; Hock et al. 2019), this side effect should also be taken into account. Electrical conductance is rarely monitored in Alpine rivers; baseline levels are naturally low (< 400 μs in typical alpine valleys (Weijs et al. 2013) depending on bedrock composition), and therefore, changes appear negligible and not alarming (but see chloride monitoring in a highly anthropized northern Italian catchment (Nava et al. 2020)). However, our results call for an implementation of monitoring programs in rural and urban Alpine catchments, since the observed fluctuations might stress the biota in these rivers, which presumably are adapted to low and relatively constant conductivities (Niedrist and Füreder 2016). The management of road salt salinization in mountain areas could be considerably improved: First, the implementation of long-term surveys of discharge and conductance together with ion concentrations, especially in low-order mountain streams, are essential to assess the temporal variability in salinity at various time scale and especially capture salinization events (Timpano et al. 2018). These detailed and systematically planned surveys would serve to anticipate salinization and to forecast salinity concentrations according to different climatic and/or management scenarios. Indeed, some predictive models have already shown that a great percentage of streams will double their salt concentration in Germany (Le et al. 2019) and the USA (Olson 2019) during this century, but no models have been developed for the Alps. Second, alternative deicers with a lower impact on aquatic biodiversity should also be considered (Breen 2017). Also, calibrating the amount of salt needed by surface area to assure driving safety and improving the equipment accuracy could greatly reduce salinization in mountain streams (Kelly et al. 2010).

## Conclusions

The analysis of multi-year electrical conductance time series, obtained by the environmental agency of Tyrol (Hydrographischer Dienst Tirol), provides evidence that ion concentrations in Alpine streams peak during the end of winter and underline the importance of continuous and high-frequent salinity measurements in Alpine rivers. The changes in electrical conductance in winter during long and constant low-flow periods, especially in smaller catchments with low pollutant dilution capacity (Williams and Melack 1997; Corsi et al. 2015), and considerable urban infrastructures, suggested that it could be related to the application of deicing road salt. Thus, in small streams draining catchment with urban surface areas or located close to deicing zones (streets, parking lots, settlements), aquatic organisms could be exposed to short-term concentrated salt pulses in these mountain streams and can additionally be impaired by the side effects of salinization (through mobilization of other toxic substances from soils). However, the occurrence, the frequency, the intensities, and the consequences of such salinity peaks for benthic organisms are not well known (Hintz and Relyea 2019) and need to be studied in Alpine rivers.

To estimate direct inputs from roads into near running Alpine waters, we thus recommend to directly monitor salt concentrations of low-discharge Alpine streams with varying degree of road kilometers in their hydrological catchments together with discharge data for discharge corrections (Hirsch et al. 2015) and load comparisons over time (Runkel et al. 2004). Additionally, assessing behavioral effects (e.g., invertebrate drift or emergence dynamics) due to short peaks of electrical conductivity during winter, which corresponds to the dry season in mountain catchments, will broaden the understanding of salinization effects for aquatic organisms in Alpine rivers. Finally, further studies should also consider the interaction of salts with other stressors (Velasco et al. 2019), such as materials derived from traffic (e.g., fine dust, particulate inorganic matter, nitrogen oxide, rubber abrasion) that accumulate in snow next to roads during the winter and are partly transported into the rivers during spring melting events (Krein and Schorer 2000; Kaushal et al. 2017; Müller et al. 2020).

## Electronic supplementary material

ESM 1 (PDF 712 kb)

## Data Availability

Data supporting the findings are available from the corresponding author upon reasonable request.
