# Supplementary material for: Salinization of Alpine rivers during winter months
Source: Environ Sci Pollut Res Int. 2020 Oct 7;28(6):7295–306. doi: 10.1007/s11356-020-11077-4 (PMC7840655; doi:10.1007/s11356-020-11077-4)
Supplement: Supplementary file 1 — (PDF 712 kb) [file 11356_2020_11077_MOESM1_ESM.pdf]

Salinization of Alpine rivers during winter months

Georg H. Niedrist[1]*, Miguel Cañedo-Argüelles[2] and Sophie Cauvy-Fraunié[3]

[1] River and Conservation Research, Department of Ecology, University of Innsbruck, Austria
[2]Freshwater Ecology, Hydrology and Management group (FEHM), Departament de Biologia Evolutiva, Ecologia i Ciències Ambientals, Institut de Recerca de l'Aigua (IdRA), Universitat de Barcelona, Barcelona, Spain
[3]INRAE, UR RIVERLY, Centre de Lyon-Villeurbanne, Villeurbanne Cedex, France

*** Corresponding author: e-mail: Georg.Niedrist@uibk.ac.at, tel.: +43 650 3124871**

Supplementary figures

The following figures complement the findings on the salinization of Alpine rivers. In particular, the illustrations of conductance and discharge trends (without seasonal patterns and random fluctuations) and of deviations in the general relationships between discharge and conductance within the studied sites support the statements in the publication.

Fig. S1. Multi-year trend (12-month moving average) of specific conductance (black) and discharge (blue) at site A, the river Sanna, Tyrol, Central Alps, with indications of significant changes over the study period. n.s. = not significant. Ticks at the x-axis mark Jan 1st.

Fig. S2. Time-series of specific conductance in different temporal resolutions, ranging from 15min-intervals to monthly data (indicated in the individual figure panels). Colored polygons delineate ‘summer’ (in red) and ‘winter’ (in blue) seasons.

Fig. S3. Electrical conductance and discharge in study sites A, B, C, and D from September 2018 to May 2019. Blue areas mark periods, where winter tires are mandatory for cars in the region (Nov 1 until April 15, “W”). Black lines are hourly conductivity data, blue lines are daily discharge data (second y-axis). Arrows indicate focus areas to illustrate the relationship between conductivity and discharge (negative as in D) or irregularities of that (e.g., in sites A and C).

Fig. S1

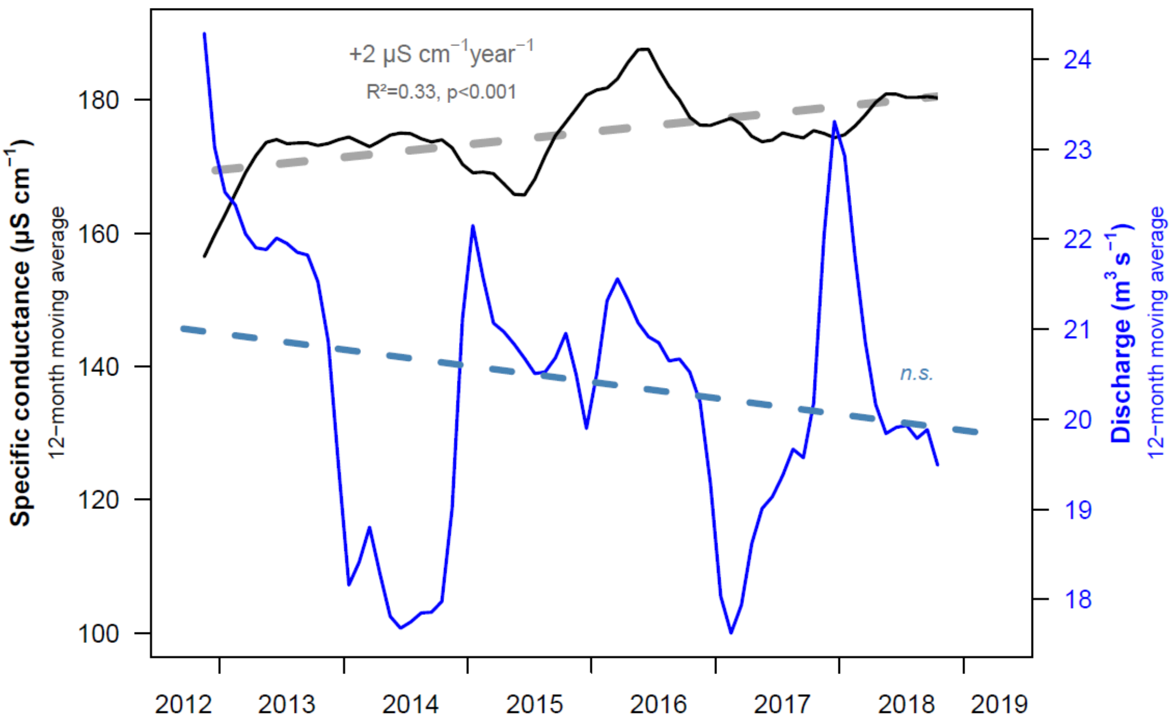

Fig. S1. Multi-year trend (12-month moving average) of specific conductance (black) and discharge (blue) at site A, the river Sanna, Tyrol, Central Alps, with indications of significant changes over the study period. n.s. = not significant. Ticks at the x-axis mark Jan 1st.

Fig. S2

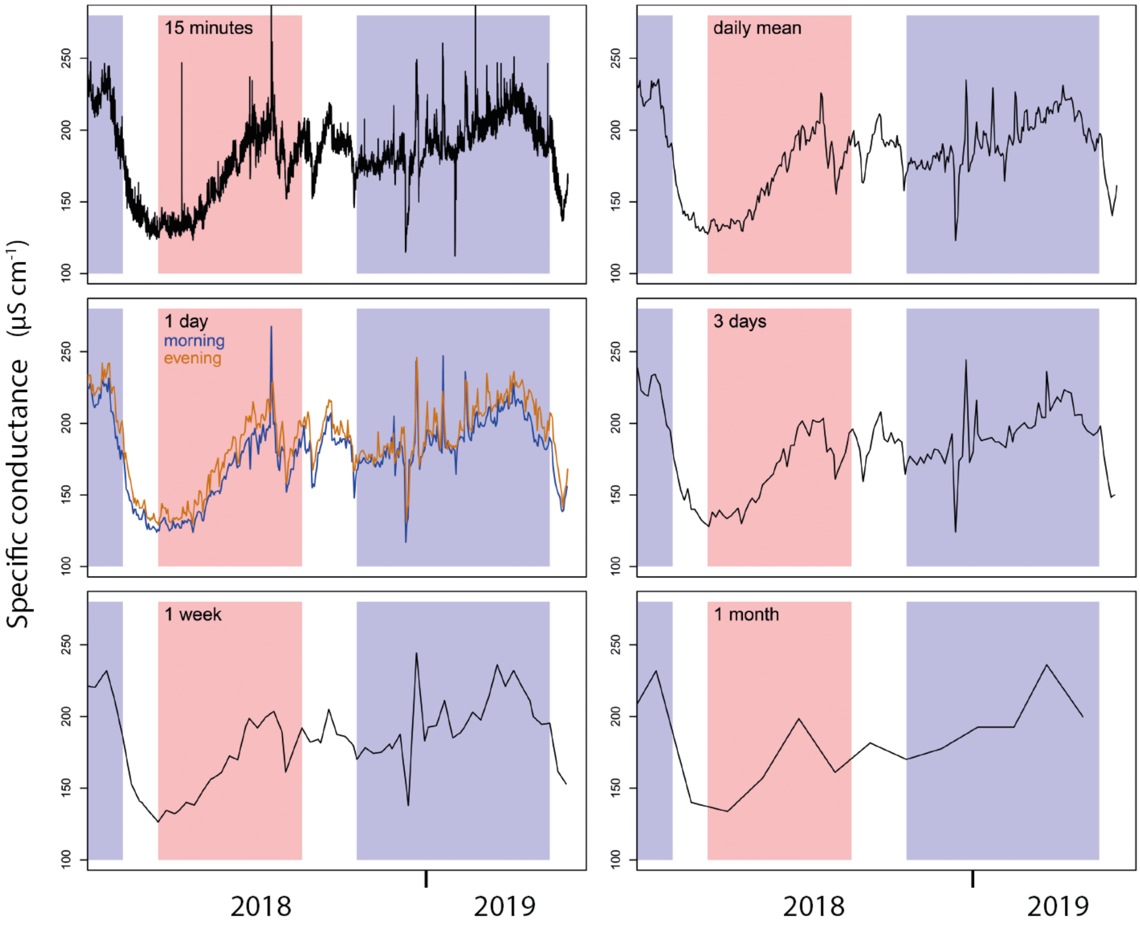

Fig. S2. Time-series of specific conductance in different temporal resolutions, ranging from 15min-intervals to monthly data (indicated in the individual figure panels). Colored polygons delineate ‘summer’ (in red) and ‘winter’ (in blue) seasons.

Fig. S3

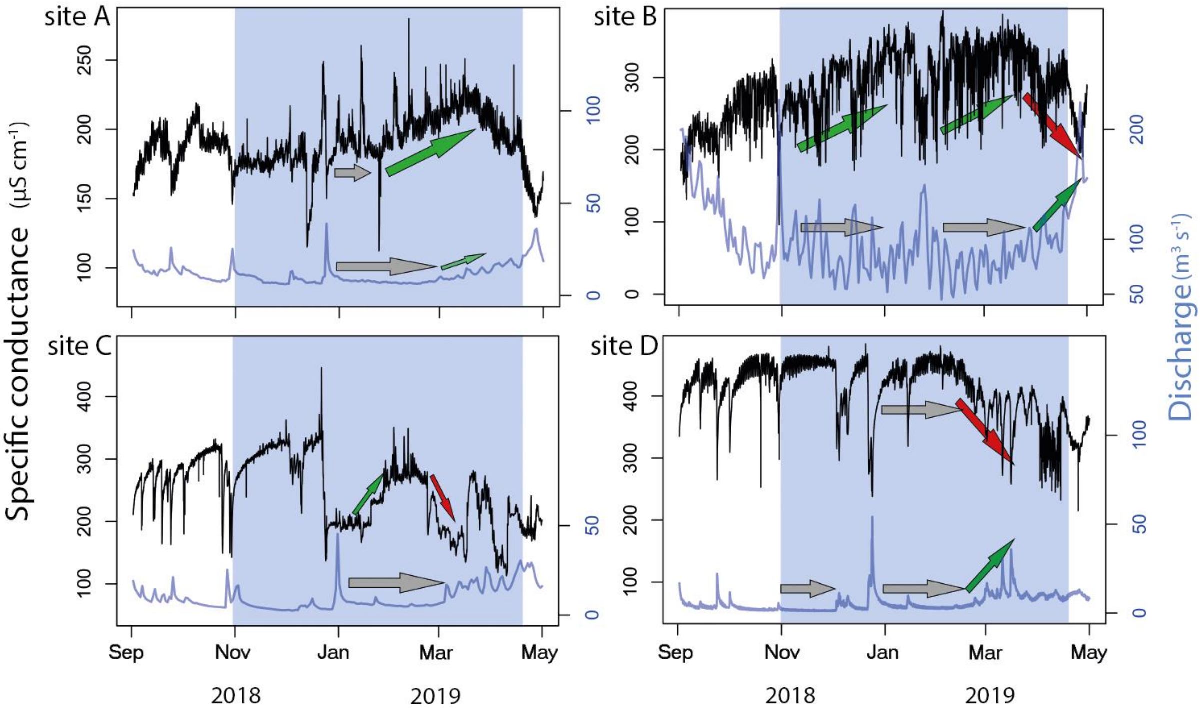

Fig. S3. Electrical conductance and discharge in study sites A, B, C, and D from September 2018 to May 2019. Blue areas mark periods, where winter tires are mandatory for cars in the region (Nov 1 until April 15, "W"). Black lines are hourly conductivity data, blue lines are daily discharge data (second y-axis). Arrows indicate focus areas to illustrate the relationship between conductivity and discharge (negative as in D) or irregularities of that (e.g., in site A and site C).
